# Supplementary material for: Spatial defects nanoengineering for bipolar conductivity in MoS2
Source: Nat Commun. 2020 Jul 10;11:3463. doi: 10.1038/s41467-020-17241-1 (PMC7351723; doi:10.1038/s41467-020-17241-1)
Supplement: Supplementary file 1 — Supplementary Information [file 41467_2020_17241_MOESM1_ESM.pdf]

Supplementary Information

Spatial Defects Nanoengineering for Bipolar Conductivity in MoS_2

Xiaorui Zheng[#], Annalisa Calò[#], Tengfei Cao[#] et al.

Supplementary Note 1: Temperature at the tip-sample contact.

The heat efficiency parameter *C*, defined as the ratio between the rise of the surface temperature T_{int} and the rise of the heater temperature T_{Heater}, is expressed as[1]:

$$C = \frac{T_{int} - RT}{T_H - RT} = \frac{R_{spread} + R_{ms-int}}{R_{tip} + R_{tm-int} + R_{spread} + R_{ms-int}} \quad (1)$$

Where RT is the room temperature, R_{spread} is the spread thermal resistance and R_{ms-int} is the thermal resistance at the MoS_2/SiO_2 interface, which we assume >> R_{spread} in case of monolayer MoS_2 exfoliated on a SiO_2 substrate[2]. Under this assumption, the expression for C can be simplified to:

$$C = \frac{T_{int} - RT}{T_H - RT} = \frac{R_{ms-int}}{R_{tip} + R_{tm-int} + R_{ms-int}} \sim 0.45 \quad (2)$$

R_{tm-int}, the thermal resistance at the tip-MoS_2 interface is usually dependent on the nanoscale morphology of the contact and on pressure. In this model, we treat it as a single asperity so that:

$$R_{tm-int} = {r_{int}}/{\pi a^2} = 2.6 \cdot 10^6\ KW^{-1} \quad (3)$$

Where $r_{int} = 10^{-8}\ W^{-1}Km^{2}$[2] and a = 35 nm is the radius of the tip.

$$R_{ms-int} = {1}/{TBC_{int}\, \pi a^2} = 1 \cdot 10^7\ KW^{-1} \quad (4)$$

Where $TBC_{int} = 26 \cdot 10^6\ WK^{-1}m^{-2}$ is the thermal boundary conductance for the MoS_2/SiO_2 interface[3], and a = 35 nm is the radius of the tip.

R_{tip}, the thermal resistance of the tip, can be estimated from the thermal conductivity of silicon nanowires, and is of the order of $10^7\ KW^{-1}$[2].

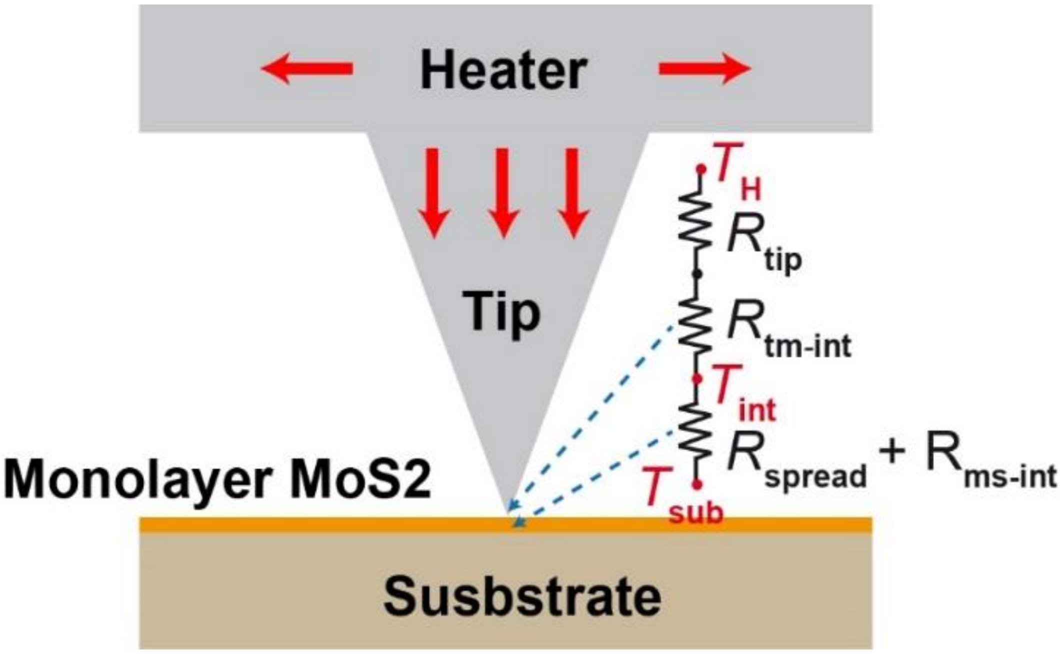

Supplementary Figure 1. Thermal circuit model. Thermal circuit for the heat flow of the system comprising a heated tc-SPL thermal cantilever, the tip and a MoS_2 monolayer flake positioned on a substrate.

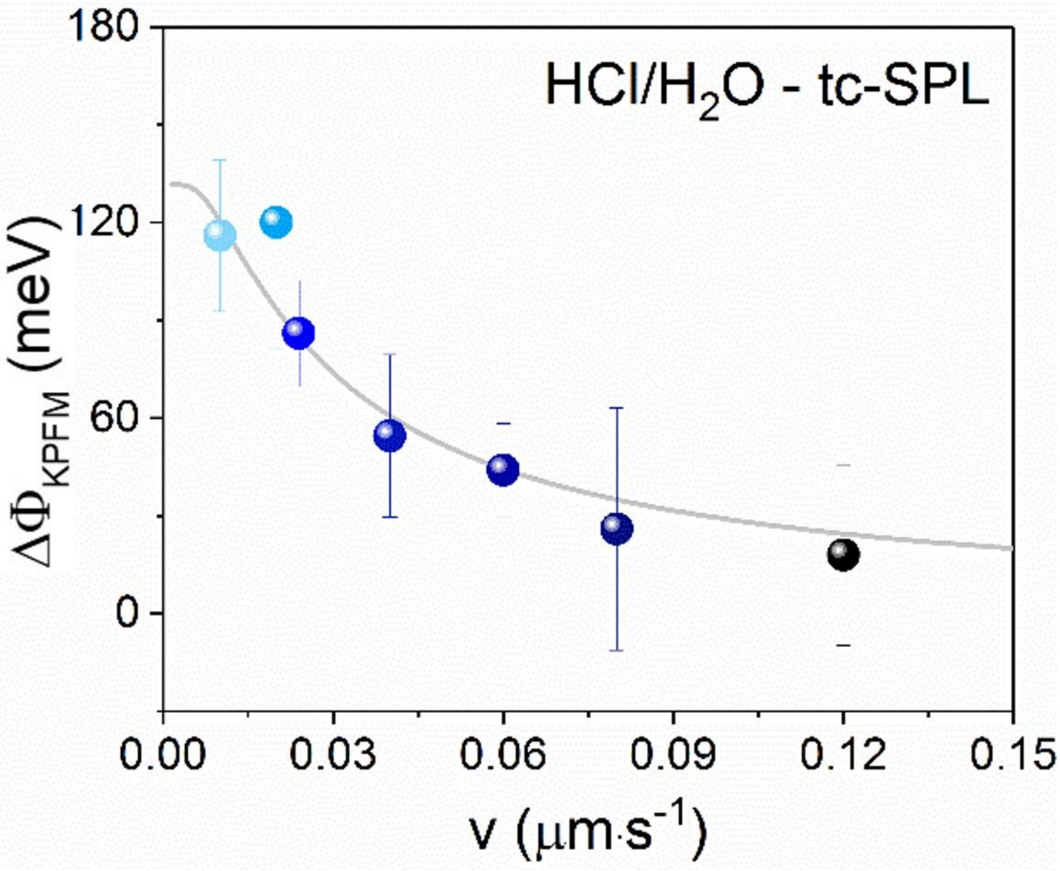

Supplementary Figure 2. First order kinetic model for tc-SPL in HCl/H_2O atmosphere. Plot of the work function difference between the tc-SPL pattern and the unheated MoS_2 flake $\Delta\Phi_{KPFM} = \Phi_{pattern} - \Phi_{non\text{-}patterned}$ vs. scan rate (*v*). Data fit (line in grey) is performed according to the Equation: $\Delta\Phi = \Delta\Phi_{pattern} - \Delta\Phi_{non-pattern} = \Delta\Phi_0 \cdot (1 - e^{-\frac{A \cdot r}{v} \cdot e^{-\frac{E_a}{RT}}})$, from which E_a = 2.03 eV is obtained by setting A = 10^{12} s^{-1}, r = 70 nm and T = 823 K (R^2 = 1).

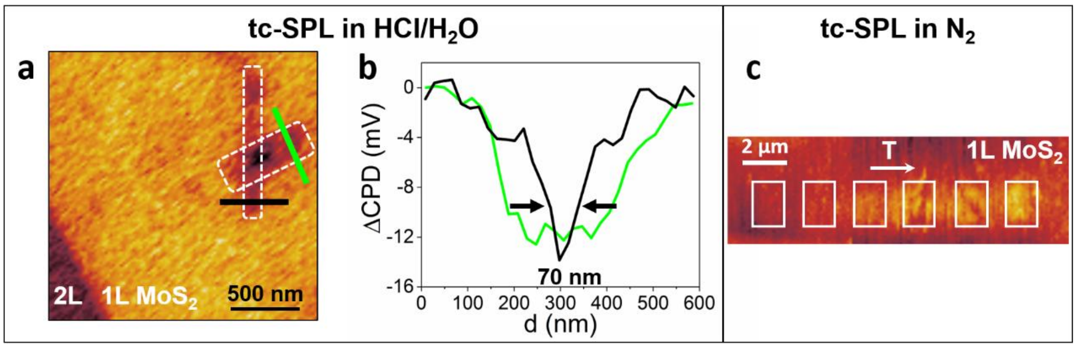

Supplementary Figure 3. tc-SPL doping. (**a**) Amplitude-modulated Kelvin Probe Force Microscopy (AM-KPFM) image of a monolayer exfoliated MoS_2 where two rectangular regions have been patterned by tc-SPL in HCl/H_2O atmosphere at different scan angles (scan rate: 0.04 $\mu m \cdot s^{-1}$, T_{Heater} = 1473 K, resolution: 8 nm/line and deflection setpoint: 6 V). Z-scale: 40 mV. (**b**) Contact potential difference profile ΔCPD ($CPD_{pattern}$ - $CPD_{non\text{-}patterned}$) corresponding to the cross section along the two lines in black and in red shown in (**a**). The distance (d) corresponding to the full width at half maximum is 70 nm for the smallest feature (vertical rectangle in (**a**)) and 270 nm for the larger rectangle in (**a**). (**c**) AM-KPFM image of a single layer CVD monocrystalline MoS_2 where micrometric sized rectangular regions have been patterned by tc-SPL in N_2 atmosphere (scan rate: 0.2 µm/s, resolution: 3.9 nm/line, deflection setpoint ~ 3V) at six different temperatures (T_{Heater} = 822 K, 913 K, 1004 K, 1035 K, 1065 K and 1097 K). Z-scale: 100 mV.

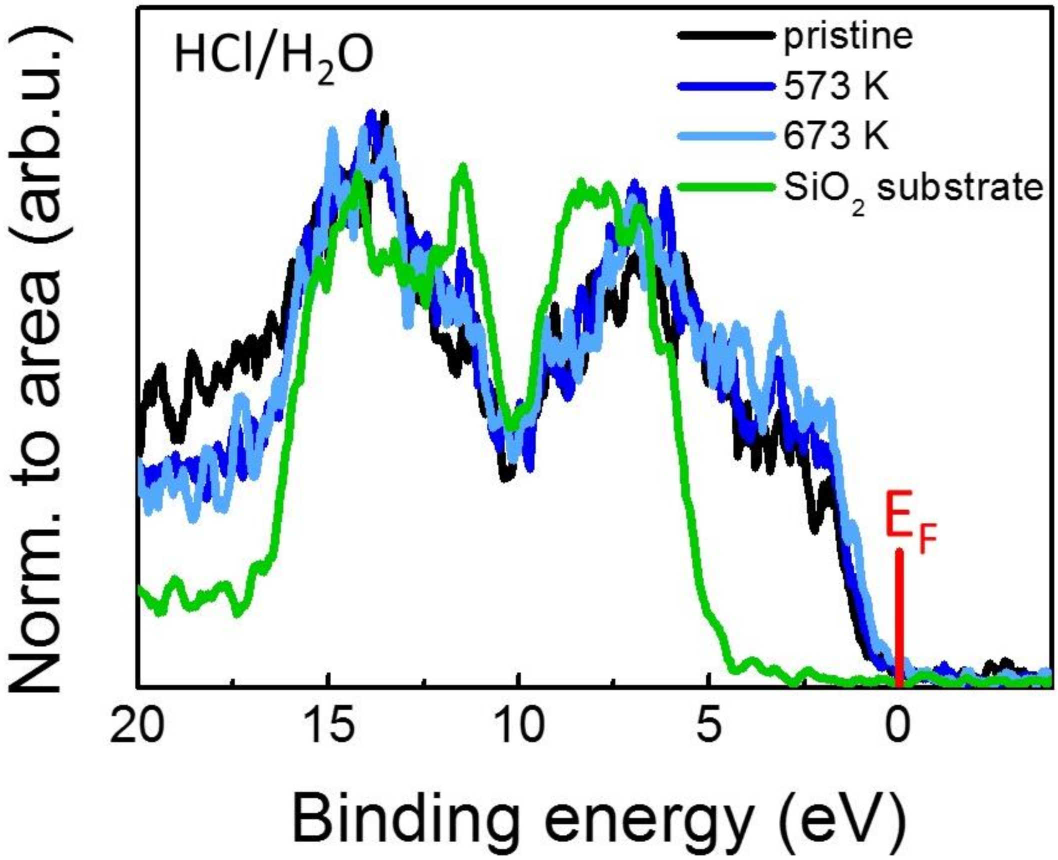

Supplementary Figure 4. XPS analysis. Valence band (VB) of the MoS_2 exfoliated samples heated in HCl/H_2O atmosphere at two different T (573 K and 673 K) compared with the pristine MoS_2. The bare SiO_2 substrate is also shown (green curve). For a better comparison the VB spectra are normalized to the area calculated in the energy range of the plot. Close to the Fermi level we can notice that the substrate does not affect the shift of the valence band maximum of MoS_2 samples.

Supplementary Note 2: Comparison of work function shifts obtained in DFT, XPS, KPFM and FET measurements

Regarding the comparison of work function shifts obtained in FET measurements with the XPS results, we remark that the work function is the energy difference between the vacuum level and Fermi energy, while the highest energy level of the valence band (VBM) is measured during the XPS experiments (VBM_{XPS}) as the energy difference, considered positive, between the Fermi energy and VBM. In particular, for n-doping we measured $\Phi_{n\text{-type}} - \Phi_{as\text{-grown}} = \Delta\Phi = -18$ meV and $\Delta VBM_{XPS} = VBM_{XPS}$ (n-type) - VBM_{XPS} (as-grown) = +250 meV. These two energy shifts are in agreement since an upward shift in the Fermi energy corresponds to a decrease in the work function and a corresponding increase in VBM_{XPS}, however, the two shifts are not the same in absolute value because during the n-doping process both the VBM and the Fermi level change.

Regarding the comparison of these values in p-doping case, we obtained the following. As shown in Fig. 20, first, we remark that the work function (Φ) is the energy difference between the vacuum level and Fermi energy, while the VBM measured during the XPS experiments (VBM_{XPS}) is the energy difference, considered positive, between the Fermi energy, E_{FE}, and highest energy level of the valence band; therefore, a downward shift in the Fermi energy corresponds to an increase in the work function and a corresponding decrease in VBM_{XPS}. On the other hand, the DFT simulations calculate the highest energy level of the valence band from the vacuum level, E_{vac}, (which is assumed to be at zero energy), and we call this value $VBM_{DFT.}$ Therefore, the different energy shifts measured in KPFM/FET, XPS, and DFT are related by the following relationship (see also Fig. 20):

$$VBM_{DFT} = E_{vac} – VBM = (E_{vac} – E_{FE}) + (E_{FE} – VBM) = \Phi + VBM_{XPS}$$

Now considering the differences between the energy shifts for the as grown samples compared to the tc-SPL p-doped samples, we obtain:

$$\Delta VBM_{DFT} = \Delta\Phi + \Delta VBM_{XPS}$$

When introducing the results obtained in our work, we have $\Delta\Phi = (+90 \pm 6)$ meV as discussed in the main text, and $\Delta VBM_{XPS} = VBM_{XPS}$ (p-type) - VBM_{XPS} (as-grown) = -300 meV as obtained from Fig. 2b in the main text. Regarding the DFT simulations, in the main text we have described three structure models, MoS_2 with one S vacancy and one surface S-S protruding bond (1.6% defect density), MoS_2 with one S vacancy and two surface S-S protruding bond (3.3% defect density); and MoS_2 with one S vacancy and three surface S-S protruding bond (5% defect density). If compared with a pristine monolayer MoS_2 we obtain, respectively for these structures, the following shifts: ΔVBM_{DFT} = -150 meV, -360 meV, and -550 meV. Since XPS elemental analysis shows a S/Mo ratio of 2.005, which remains constant throughout the doping procedure, we conclude that the majority of the defects produced during the p-doping process consists of one S vacancy and one surface S-S protruding bond, as also observed in the STEM

experiments. We therefore obtain:

$$\Delta VBM_{DFT} = -150 \text{ meV vs. } -210 \text{ meV} = \Delta\Phi + \Delta VBM_{XPS},$$

which we consider a good agreement, considering the limitations of this comparison.

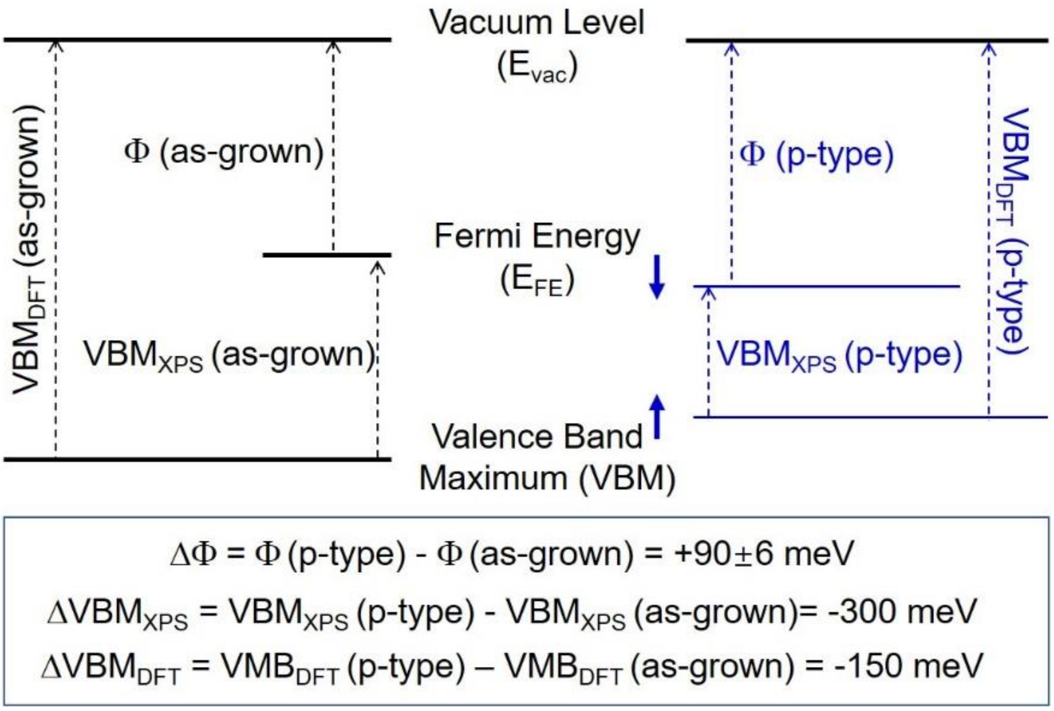

Supplementary Figure 5. **Work function comparison.** Schematic figure showing the relation of work function (Φ) measured by KPFM and FET, VBM measured by XPS (VBM_{XPS}) and the VBM_{DFT} defined in DFT calculations. The work function shift (ΔΦ), the ΔVBM_{XPS} measured in XPS, and the ΔVBM_{DFT} calculated in DFT have been listed, respectively.

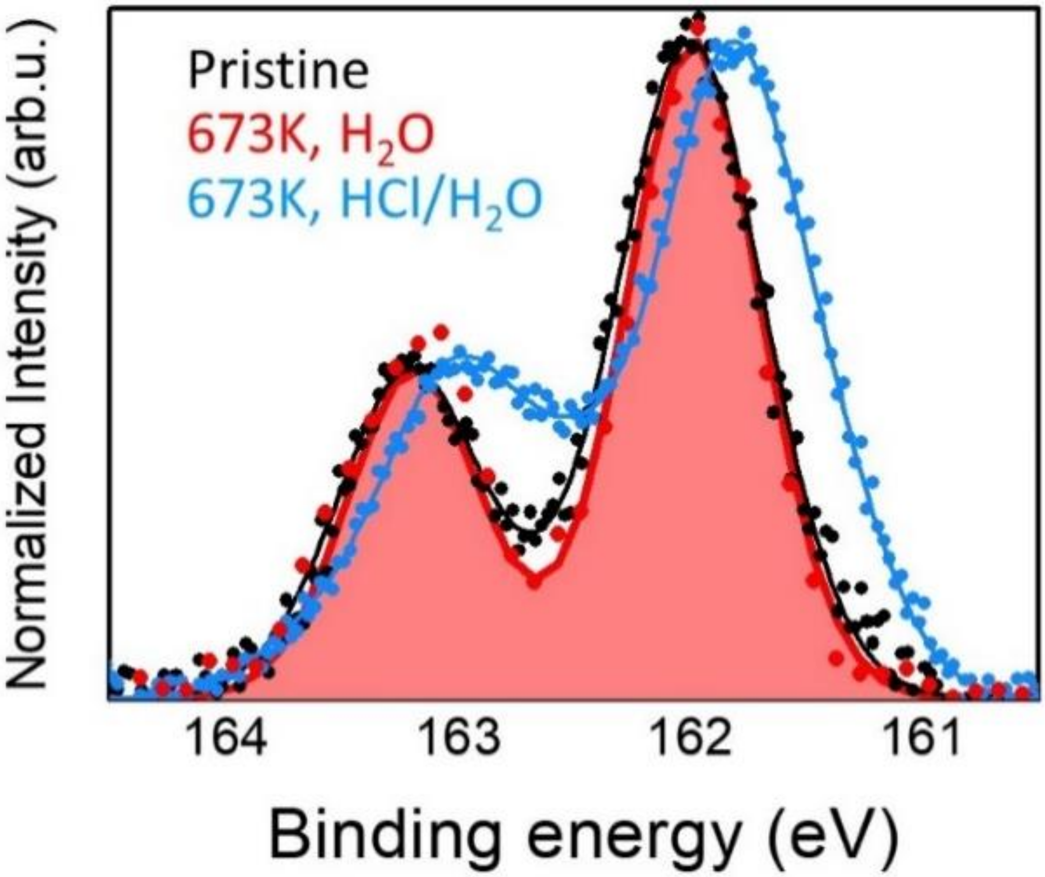

Supplementary Figure 6. S 2*p* XPS core level spectra of exfoliated MoS_2 samples. The spectrum of the pristine sample is indicated in black. The spectrum of a sample heated at 673 K for 10 minutes in humid air is indicated in red and the spectrum of the sample heated at the same temperature in HCl/H_2O atmosphere is indicated in light blue. The fit result of the sample heated in humid air, obtained with the convolution of the two 2*p*1/2-2*p*3/2 fitting components, is highlighted as red filled area. For a better comparison spectra maxima are all normalized to unity.

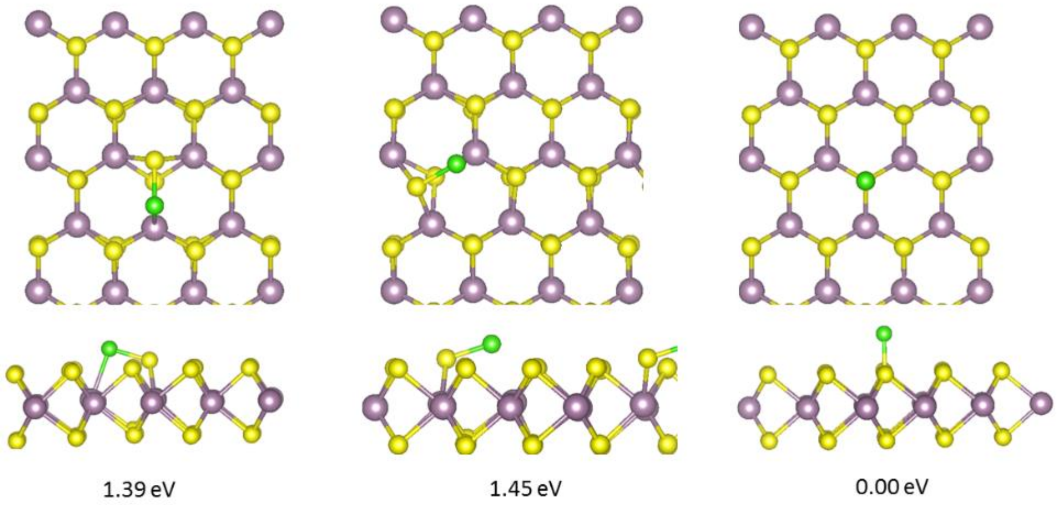

Supplementary Figure 7. Relative energy of MoS_2 with various S atoms. Relative energy of MoS_2 with S atoms adsorbed on top of a Mo atom (left structure), on top of the Mo-S bond (middle structure) and on top of a S atom (right structure).

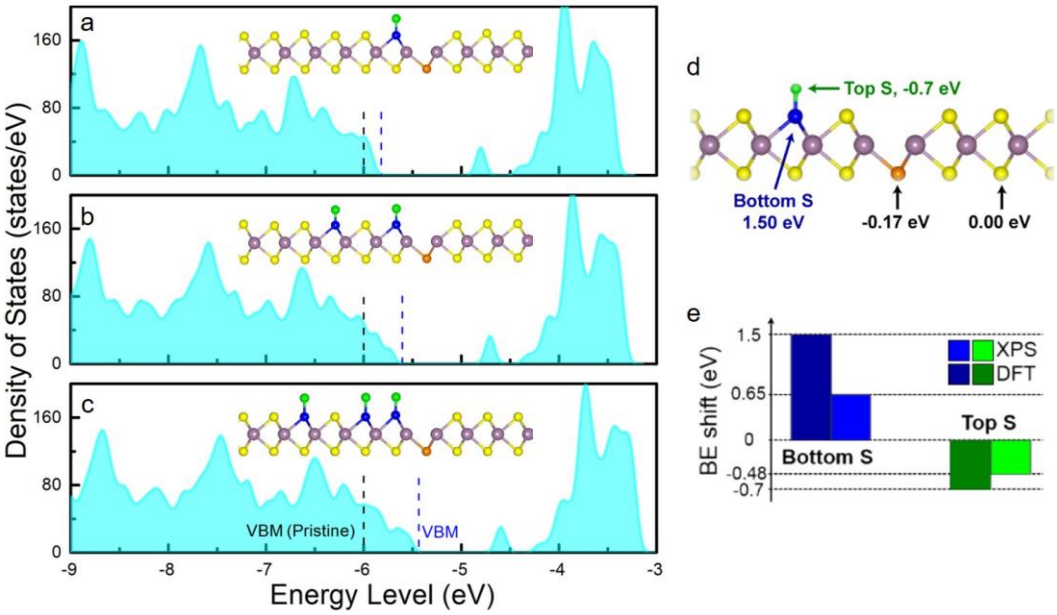

Supplementary Figure 8. DFT calculations. Density of states of MoS_2 with one S vacancy and one S (**a**), two S (**b**) and three S atoms (**c**) chemisorbed (chemically bound) on the surface. The corresponding MoS_2 structures are also given in ball and stick models: balls in violet (yellow) are Mo (S) atoms, those in green are S atoms chemisorbed on the surface (with one S below it (blue)) and ball in orange is a S vacancy position. The energy level of the valence band maximum (VBM_{DFT}) is calculated with respect to the vacuum level and it is indicated by a blue dashed line, while the VBM of pristine MoS_2 is shown with a black dashed line and obtained from Ref. [4][4]. (**d**) Side view of MoS_2 with a S vacancy and a S on top of another S atom of the matrix. The core-level energy shifts of S 2*p* states with respect to ordinary S atom (0.00 eV) are indicated. (**e**) Histogram of the DFT results (in dark blue and dark green) for the bottom and top S atoms with creation of a S vacancy together with the XPS energy shifts (in light green and light blue) of the two additional doublets used for the fit.

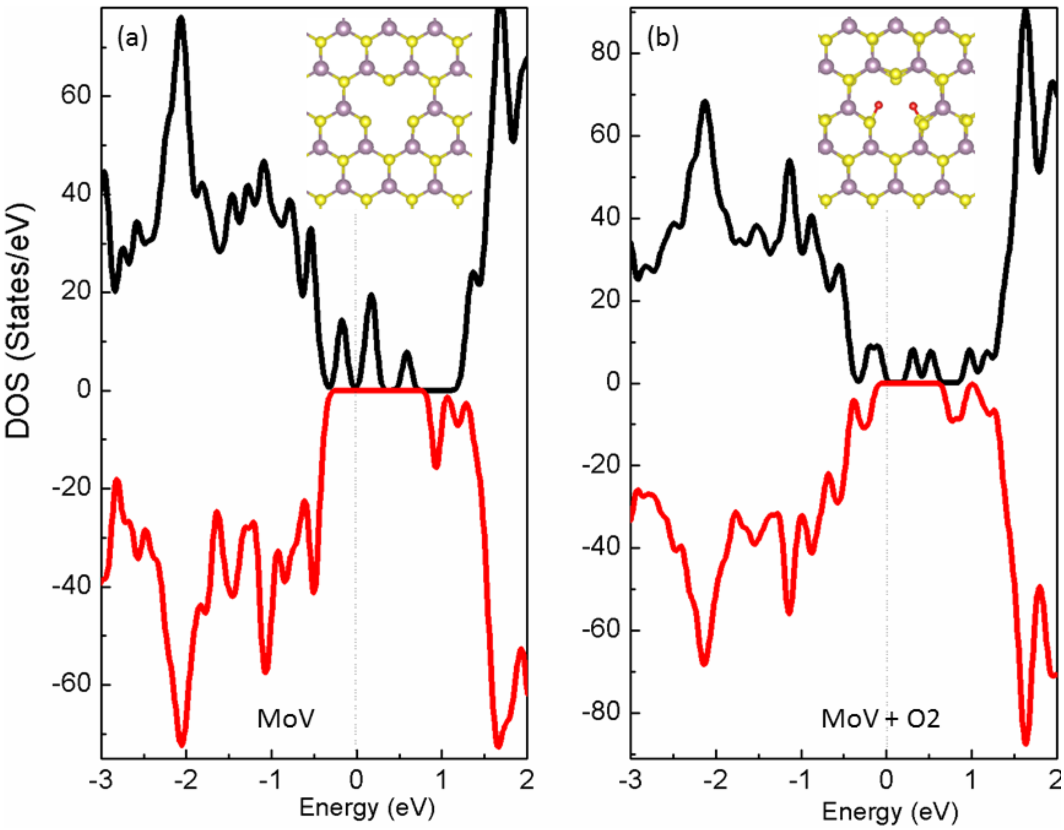

Supplementary Figure 9. DFT of Mo vacancies. Spin polarized density of states (spin-up states: black line and spin-down states: red lines) of MoS_2 with Mo vacancies (MoV) (**a**) and oxidized Mo vacancy (MoV + O_2) (**b**). Balls in brown (yellow) are Mo (S) atoms, and Oxygen atoms are indicated in red. The existence of Mo vacancy induces plenty of defect states in the band gap, and the defect states near the valence band maximum (VBM) are already occupied. Hence holes can hardly be generated in the VBM and contribute to the p-type conductivity of the sample.

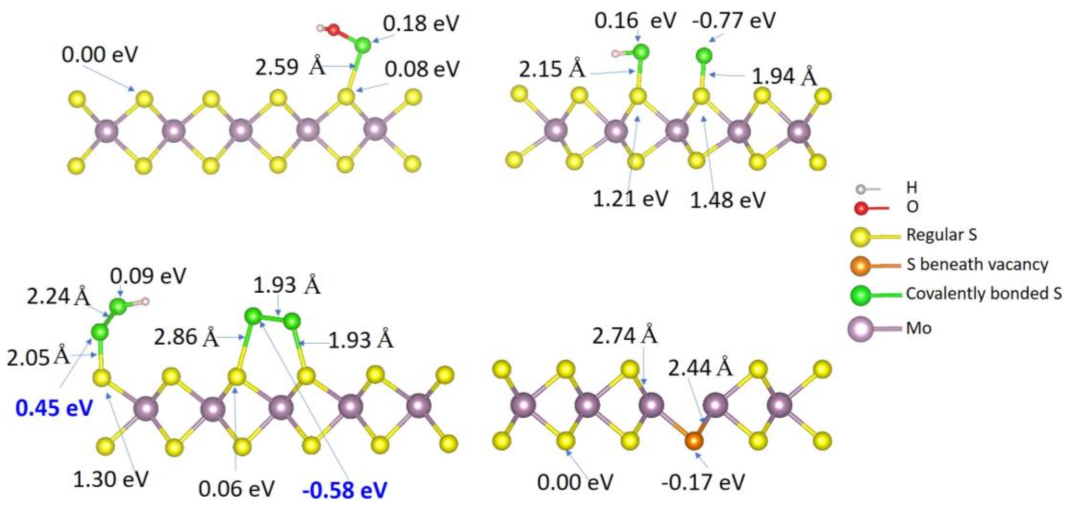

Supplementary Figure 10. Side view of adsorption states of S atom on MoS_2. The core-level energy shifts of S *2p* with respect to regular S atoms (0.00 eV) and the bond lengths around S vacancy and S dimers are indicated.

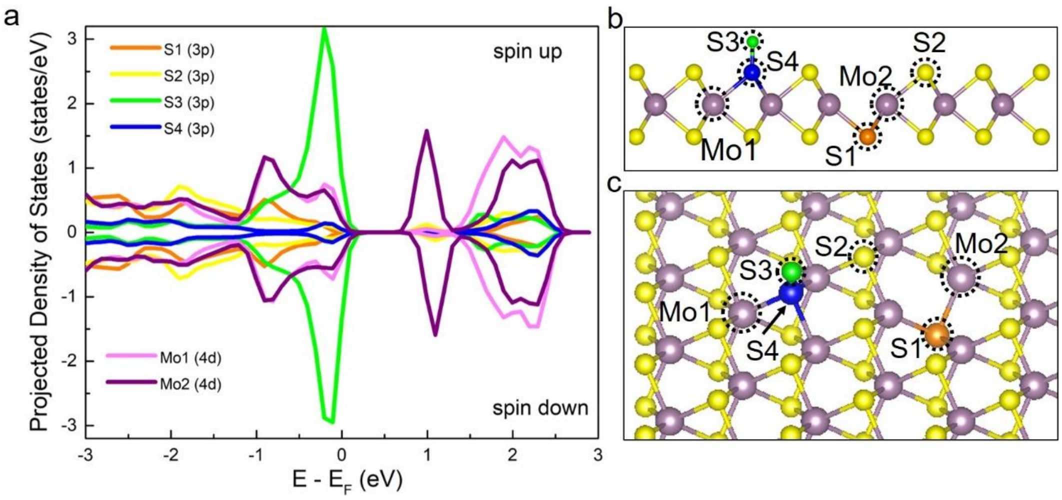

Supplementary Figure 11. Projected DOS of monolayer MoS_2. (**a**) The spin-polarized projected density of states (PDOS) of specific atoms (Mo1, Mo2, S1, S2, S3, S4) in MoS_2. The side view (**b**) and top view (**c**) of the MoS_2 atomic structures. The S atom (S1) underneath a S-vacancy, the ordinary S atom (S2), the added atom (S3) covalently bonded to a S atom on the surface (S4) are colored in orange, yellow, green and blue, respectively. Mo1 is a Mo atom bonding to S4 and Mo2 is a Mo atom bonding to S1. The PDOS proves that S3 atoms mainly contributes to the p-type character of MoS_2 observed in experiments.

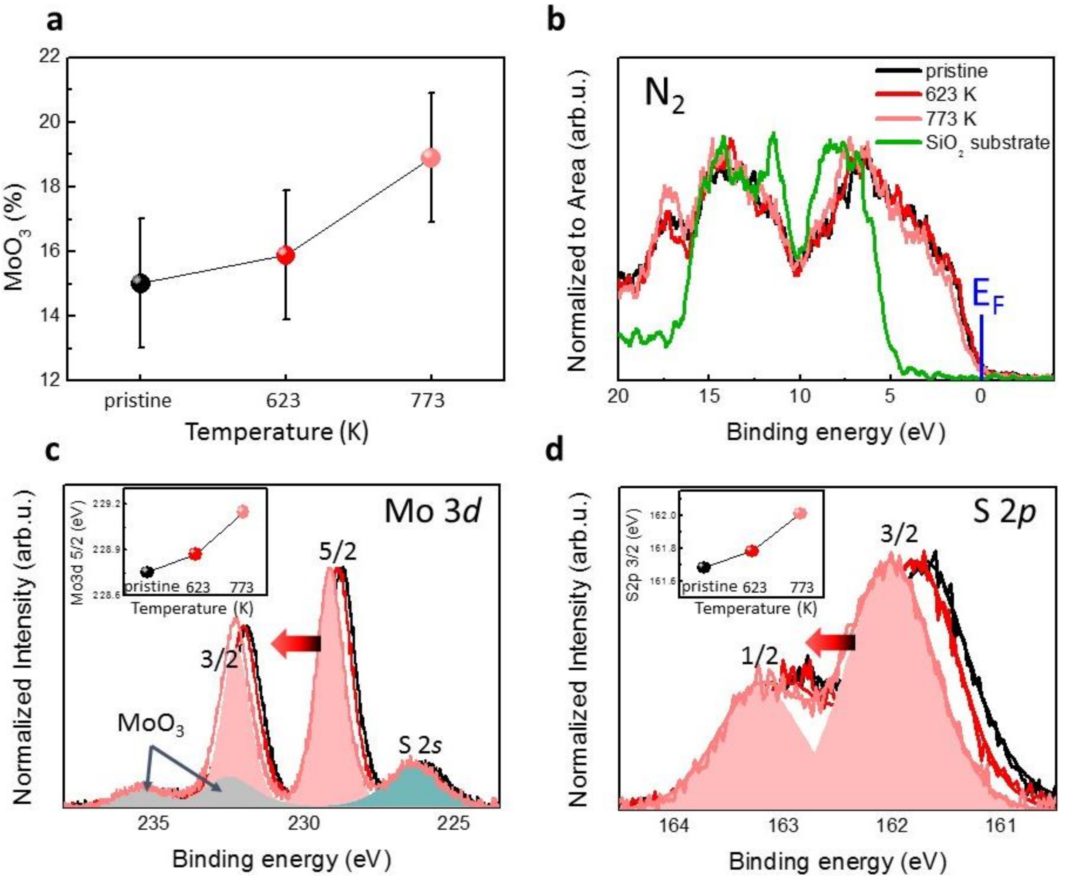

Supplementary Figure 12. XPS results of CVD MoS_2 heated in N_2. XPS results for monocrystalline single layer CVD MoS_2 samples globally heated in N_2 atmosphere at two different temperatures of 623 K (data in red) and 773 K (data in pink) compared with the pristine sample (data in black). (**a**) MoO_3 relative area in percent of the total Mo spectrum area obtained from the fit of Mo 3*d* core level spectra. (**b**) VB of the CVD MoS_2 samples compared with the bare SiO_2 substrate (green curve). VB spectra are normalized to the area calculated in the energy range of the plot. (**c**) and (**d**) Mo 3*d* and S 2*p* core level spectra, respectively. The fitting components for the sample heated at 773 K are shown. The arrows indicate the shift towards higher binding energy, in agreement with the enhanced n-doping with temperature annealing in N_2 atmosphere. The binding energy shift for the Mo 3*d*5/2 and the S 2*p*3/2 components are also shown in the insets of panel (**c**) and (**d**) respectively. For a better comparison core level spectra maxima are all normalized to unity.

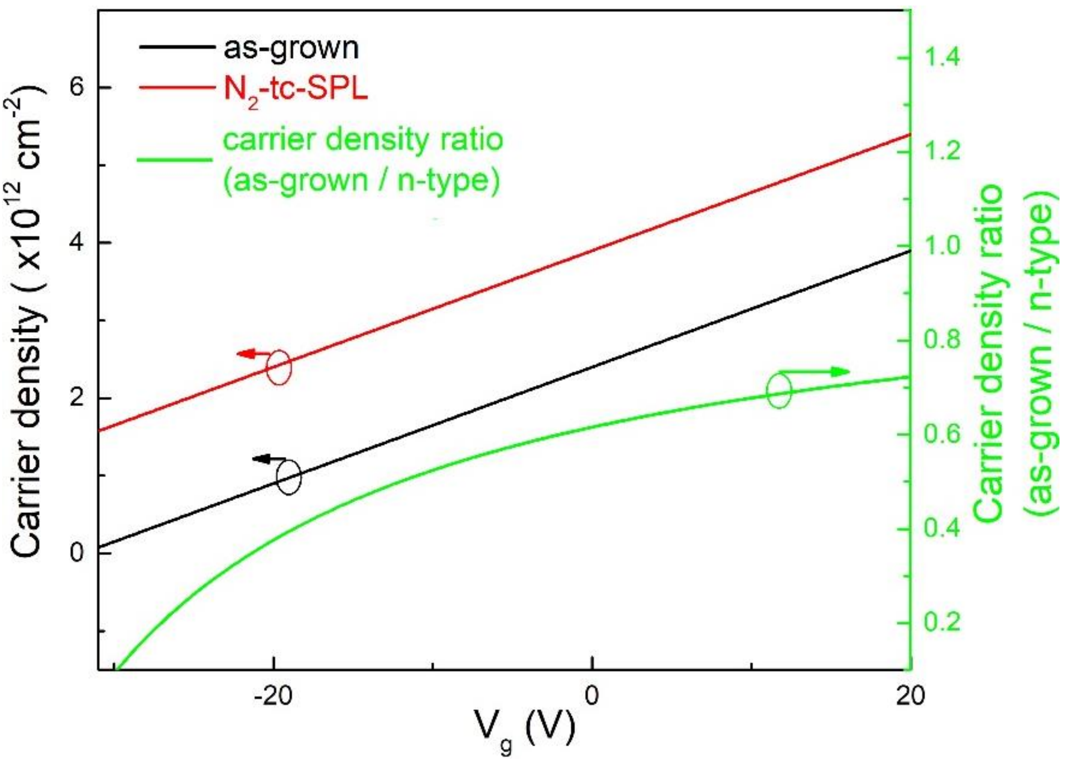

Supplementary Figure 13. Carrier density of tc-SPL n-doped FET. The carrier density before (black curve) and after (red curve) the n-type doping, and the ratio (green curve) of $\frac{n_{as-grown}}{n_{n-type}}$.

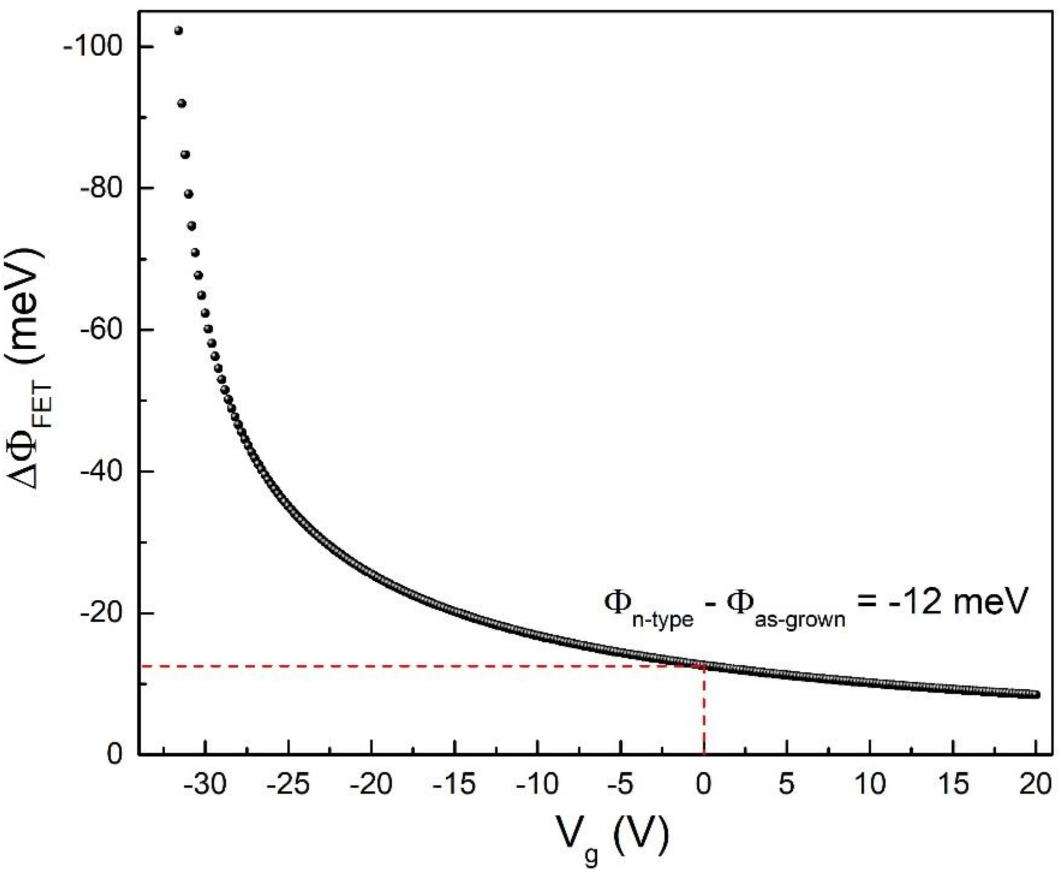

Supplementary Figure 14. Work function shift extracted from FET measurement. Plot of the FET work function shift $\Delta\Phi_{FET}$ between as-grown MoS_2 and tc-SPL n-type doped MoS_2, extracted from the electrical measurements. The work function at V_g = 0 V has been marked for direct comparison with KPFM results.

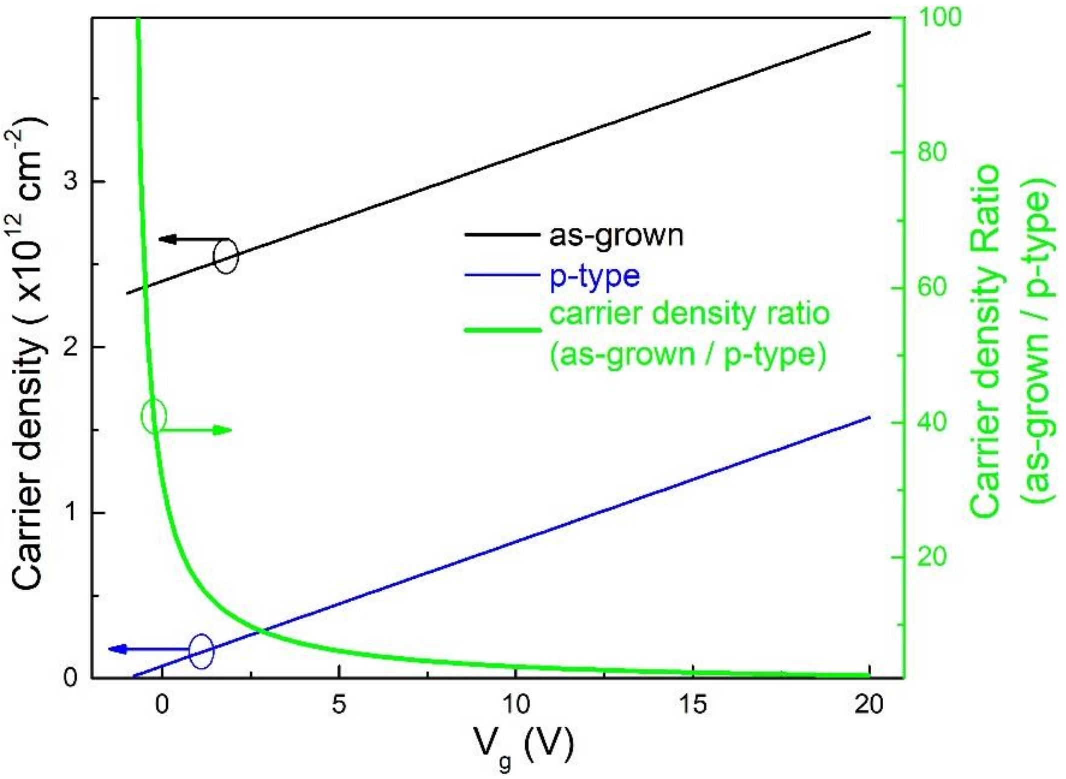

Supplementary Figure 15. Carrier density of tc-SPL p-doped FET. The carrier density before (black curve) and after (blue curve) the p-type doping, and their ratio (green curve) of $\frac{n_{as-grown}}{n_{p-type}}$.

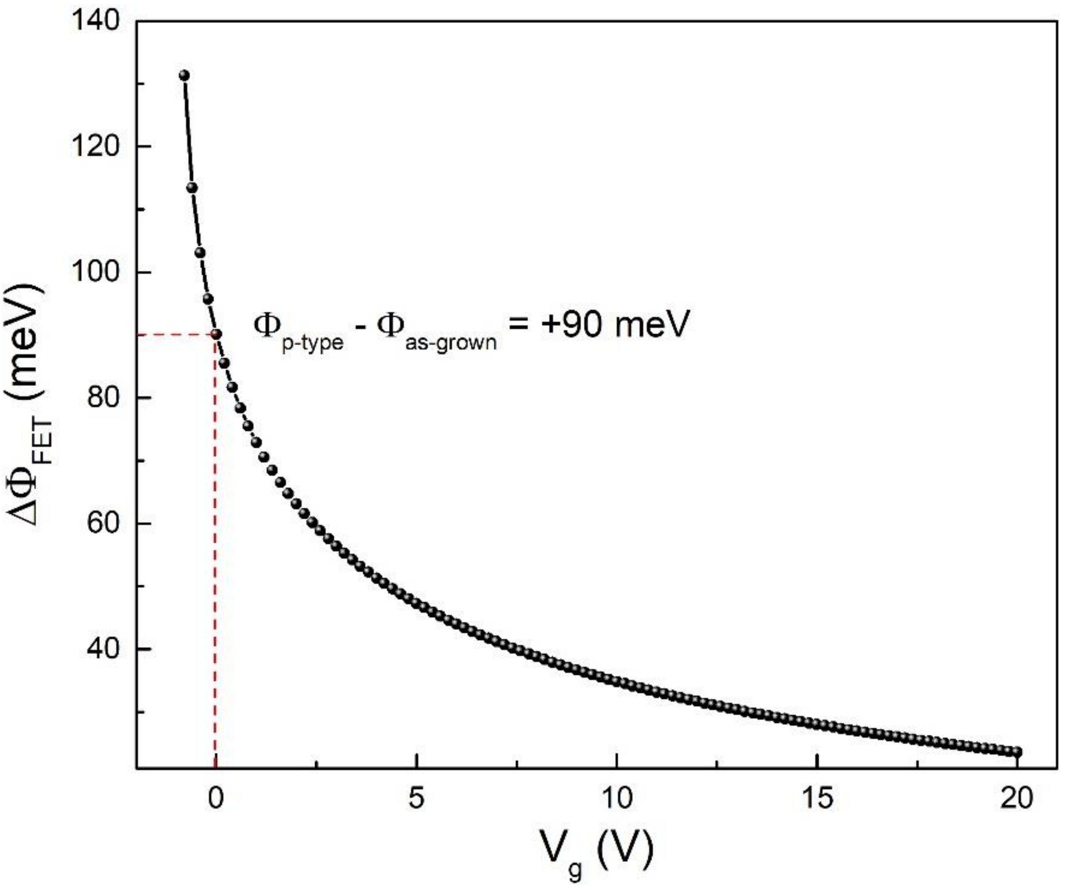

Supplementary Figure 16. Work function shift extracted from FET measurement. Plot of the FET work function shift $\Delta\Phi_{FET}$ between as-grown MoS_2 and tc-SPL p-type doped MoS_2, extracted from the electrical measurements. The work function at $V_g = 0$ V has been marked for direct comparison with KPFM results.

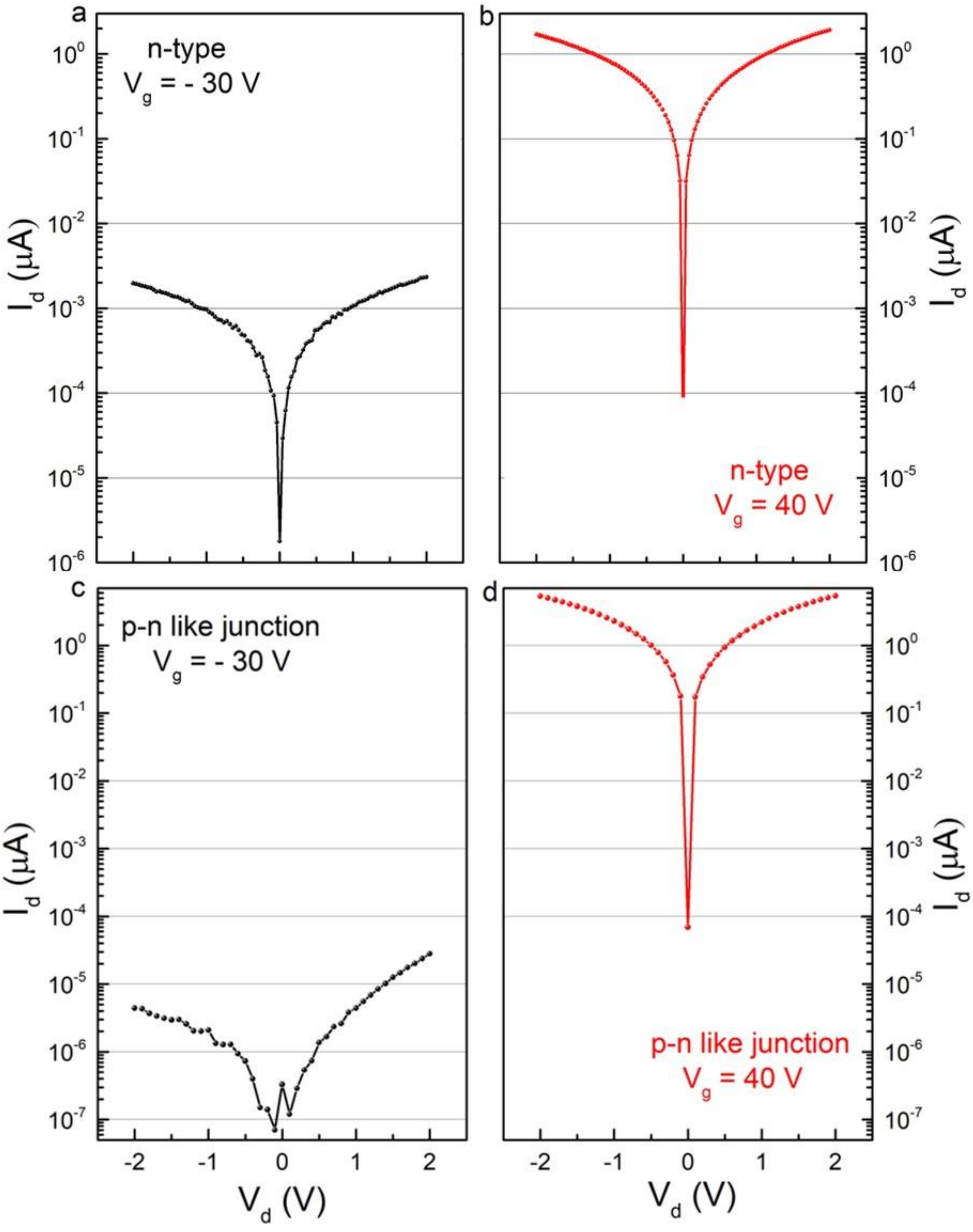

Supplementary Figure 17. Output curve comparison. (**a-b**) Output curves of the fully N_2-tc-SPL n-type FET at small (V_g = -30 V) and large (V_g = 40 V) back gating. (**c-d**) Output curves of the lateral p-n like junction at small (V_g = -30 V) and large (V_g = 40 V) back gating.

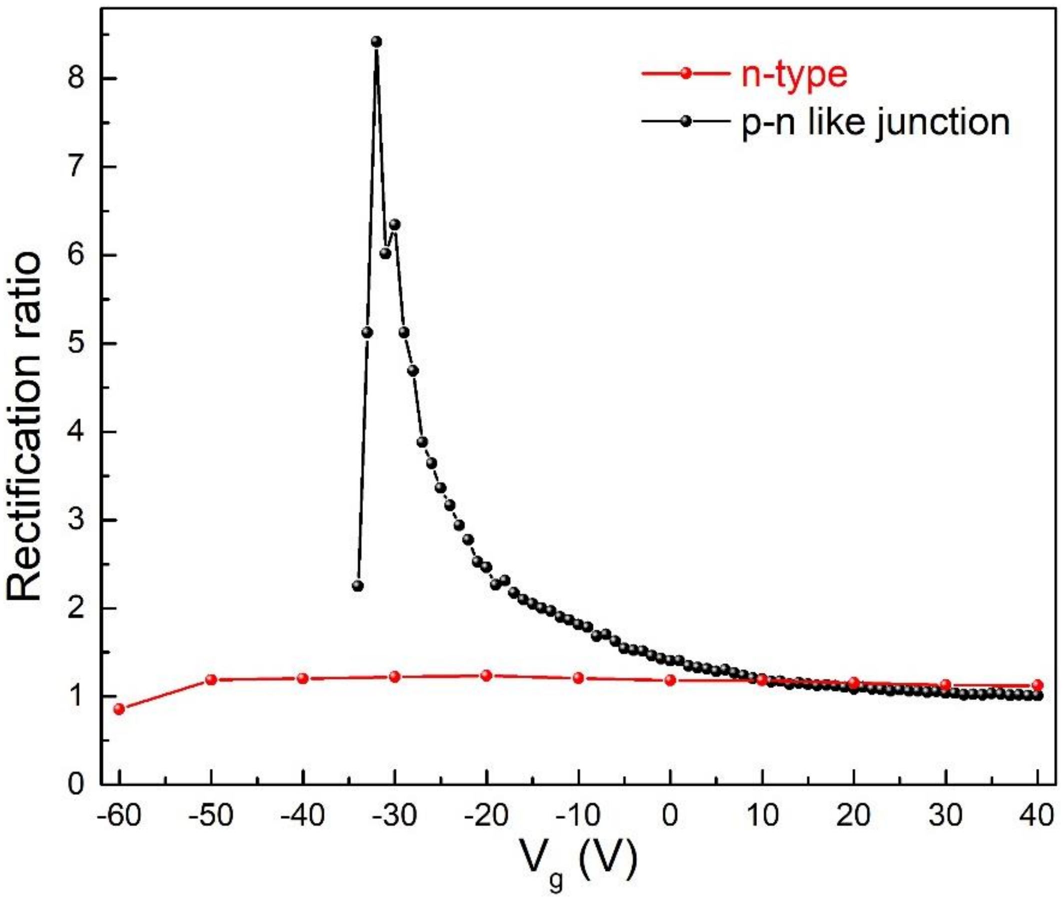

Supplementary Figure 18. Rectification ratio dependence. The rectification ratio as a function of V_g for the fully N_2-tc-SPL n-type FET (data in red) and the lateral p-n like junction (data in black).

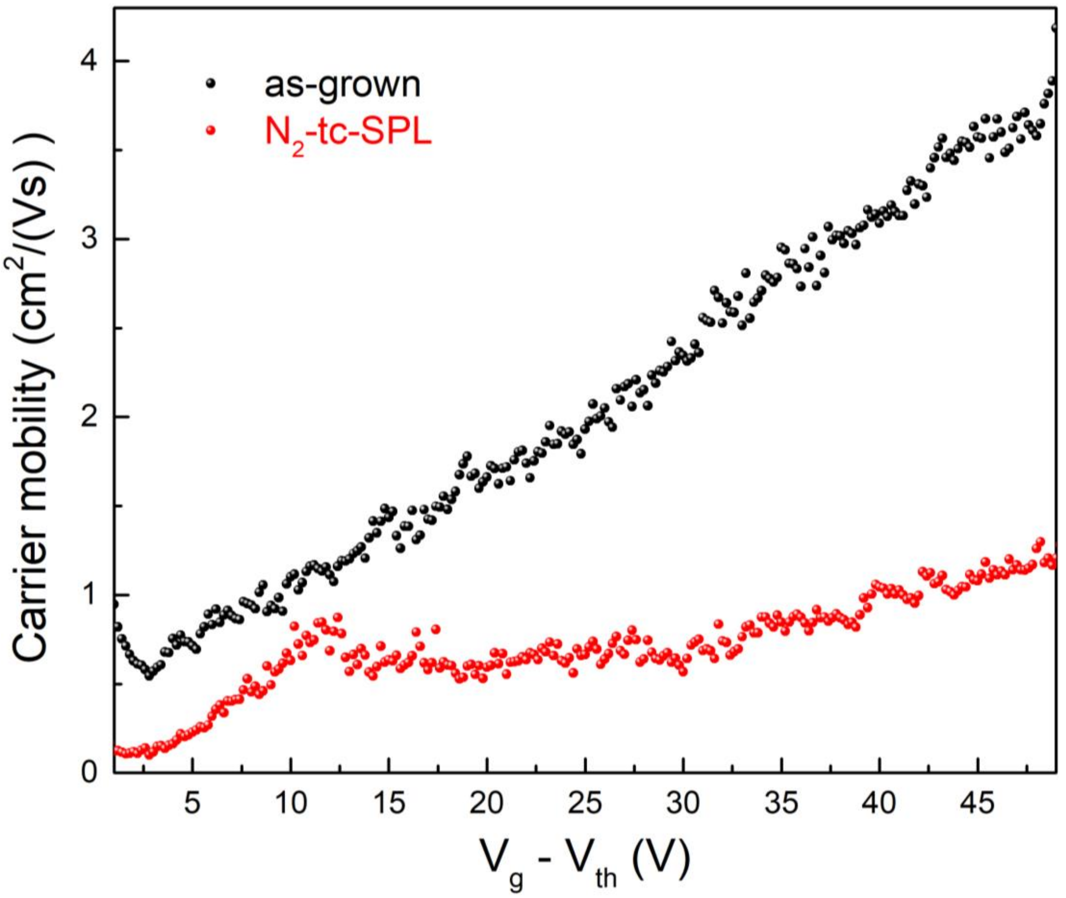

Supplementary Figure 19. Carrier mobility n-doped MoS_2. Carrier mobility of the FET before (black curve) and after (red curve) N_2-tc-SPL n-type doping of the active area.

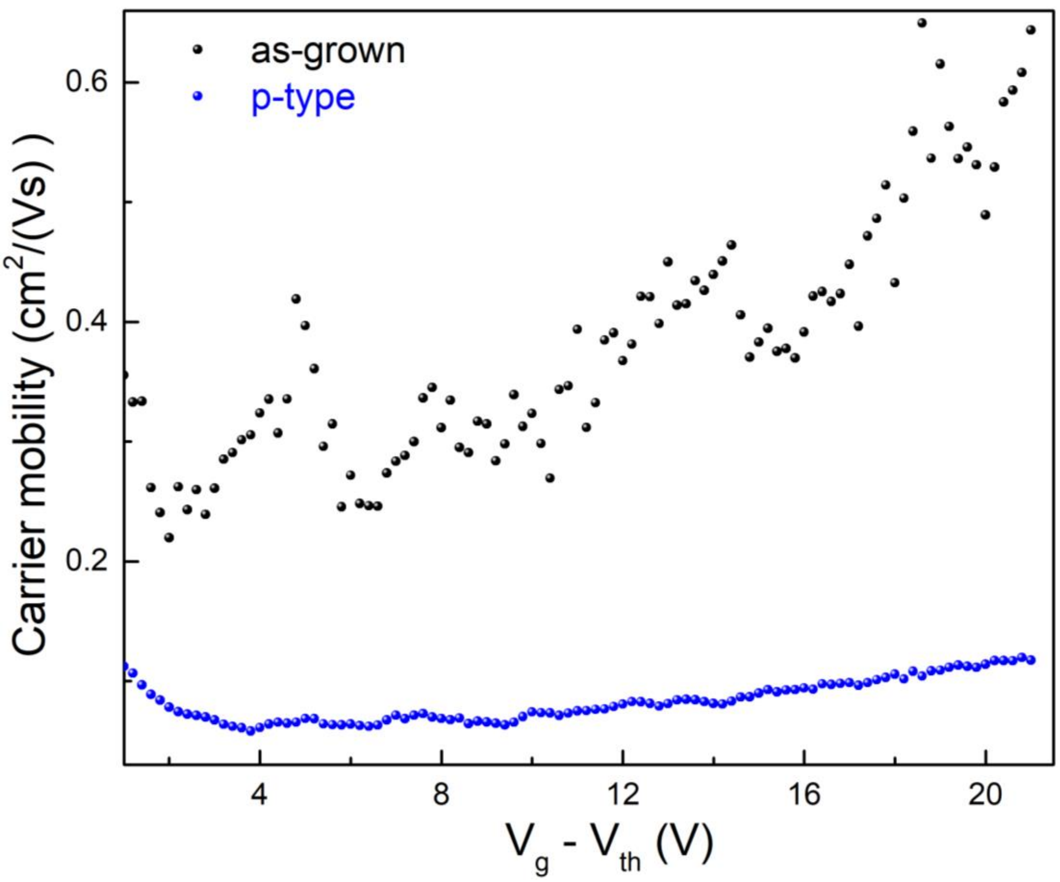

Supplementary Figure 20. Carrier mobility of p-doped MoS_2. Carrier mobility of the FET before (black curve) and after (blue curve) tc-SPL p-type doping of the active area.

Suplementary References

1 Bhushan, B. & Fuchs, H. Applied Scanning Probe Methods IV: Industrial Applications. (2006).

2 Wei, Z. *et al.* Nanoscale tunable reduction of graphene oxide for graphene electronics. *Science* **328**, 1373-1375 (2010).

3 Yasaei, P. *et al.* Interfacial thermal transport in monolayer MoS_2 - and Graphene-Based Devices. *Adv Mater Interfaces* **4**, 1700334(2017).

4 Komsa, H. P. & Krasheninnikov, A. V. Native defects in bulk and monolayer MoS_2 from first principles. *Phys. Rev. B* **91**, 125304 (2015).
